# Ultra-stable and high-performance squeezed vacuum source enabled via artificial intelligence control

**DOI:** 10.1126/sciadv.adu4888

**Published:** 2025-05-02

**Authors:** Jie Zhao, Zhifei Yu, Xin Chen, Yuan Wu, Xinyun Liang, Wenfeng Huang, Keye Zhang, Chun-Hua Yuan, L. Q. Chen

**Affiliations:** ^1^ State Key Laboratory of Precision Spectroscopy, Quantum Institute for Light and Atoms, Department of Physics and Electronic Science, East China Normal University, Shanghai 200062, China.; ^2^ School of Physics, Hefei University of Technology, Hefei, Anhui 230009, China.; ^3^ Shanghai Branch, Hefei National Laboratory, Shanghai 201315, China.

## Abstract

Squeezing states are crucial for advancing quantum metrology beyond the classical limit. Despite this, generating high-performance squeezed light with long-term stability remains a challenge due to system complexity and quantum fragility. We experimentally achieved a record-breaking squeezing level of 4.3 decibels (lossless, 5.9 decibels) using polarization self-rotation (PSR) in atomic vapor, maintaining stability for hours despite environmental disturbances. Overcoming the limitations of the PSR theory model’s optimization guidance, which arises from the mutual interference of multiple parameters at this squeezing level, we developed an artificial intelligence (AI) control (AIC) system that harnesses deep learning to discern and manage these complex relationships, thereby enabling self-adapted to external environments. This integrated approach represents a concrete step for the actual application of quantum metrology and information processing, illustrating the synergy between AI and fundamental science in breaking complexity constraints.

## Teaser

An AIC method was developed to overcome the limitations of the PSR quantum squeezing system, achieving the best performance.

## INTRODUCTION

Over the past two decades, atomic systems have become the cornerstone of many high-precision metrology applications, such as atomic clocks, atomic gravimeters, and atomic magnetometers (*1*–*9*). Quantum metrology can demonstrate performance advantages beyond classical limits (*10*–*13*), but the core of these advantages lies in the utilization of high-quality nonclassical states (*14*–*20*). Therefore, developing high-performance quantum squeezed light sources is particularly important for achieving high-precision and practical quantum metrology. To our knowledge, there have been scarce experiments (*21*, *22*) demonstrating the long-term stability and high squeezing of quantum light sources operating within the atomic absorption band.

The atom-light polarization self-rotation (PSR) coupling serves as a naturally optimal generation mechanism for quantum light sources like squeezed light in the atomic frequency band, outperforming alternatives like parametric oscillations in crystals or four-wave mixing (*23*–*27*). This is because PSR atomic systems offer the advantages of simplicity and the natural production of squeezed light within the atomic absorption band, which is ideally suited for other atomic-based quantum measurements or quantum information processing applications. In recent years, the squeezing degree and stability have been consistently improved (*28*–*32*), yet further enhancing their performance poses a formidable challenge. This arises from the fact that as research delves deeper into the strong coupling realm, achieving a perfectly precise model of the atom-light system, which is characterized by many intricately interconnected system parameters, becomes increasingly unfeasible, thereby constraining the capability to steer and guide experimental endeavors effectively. Using the PSR effect in atomic vapor to generate squeezed light is a prototypical example (*31*, *33*–*39*). In cases of a weak coupling PSR effect, the experimental results align well with a concise theoretical model that disregards higher-order nonlinear interactions and mutual influences among various atomic and optical parameters (*40*–*44*). However, upon enhancing the PSR effect to improve squeezing, the experimental outcomes substantially deviate from the model, and various attempts at improvement yield conflicting effects. Additionally, rapid jitters and slow drifts in these parameters diminish measurement stability and gradually introduce multiple noise sources, thereby degrading squeezing performance (*45*–*47*).

To address this issue, we turn to machine learning, which, through the data analysis, can directly discern optimal operational strategies within complex systems. This technique has demonstrated remarkable power across numerous domains, including successful applications in vector atomic magnetometers, ultrafast lasers, quantum error correction, parameter estimation, and others (*48*–*61*). Furthermore, for obtaining long-term stability, intelligent control of the system for adaptive response to environmental disturbances is crucial (*62*–*64*).

Here, we develop an artificial intelligence control (AIC) system and use it to experimentally achieve a stable high-performance 795-nm squeezed vacuum light via PSR in an ^87^Rb atomic vapor. Our AIC system seamlessly integrates machine learning, automatic controllers, and expert systems, which can have quick self-learning capability to find the non-analyzable relationship between squeezing degree and several core parameters without relying on PSR theoretical formulas, and intelligently control the system to achieve the optimal quantum squeezing state while maintaining long-term stability. Last, enabled by AIC, the squeezing degree is enhanced from the existing 3.0 dB (*31*) to 4.3 dB (loss rate η = 15.5%) corresponding to 5.9 dB under lossless conditions and stably maintains at this level for 2 hours with the existence of the short-term jitters and long-term drift of light intensity, frequency, atomic temperature, etc. This represents the best performance of the PSR squeezing system in the atomic absorption band up to now.

## RESULTS

### Experimental setup

**Fig. 1.**
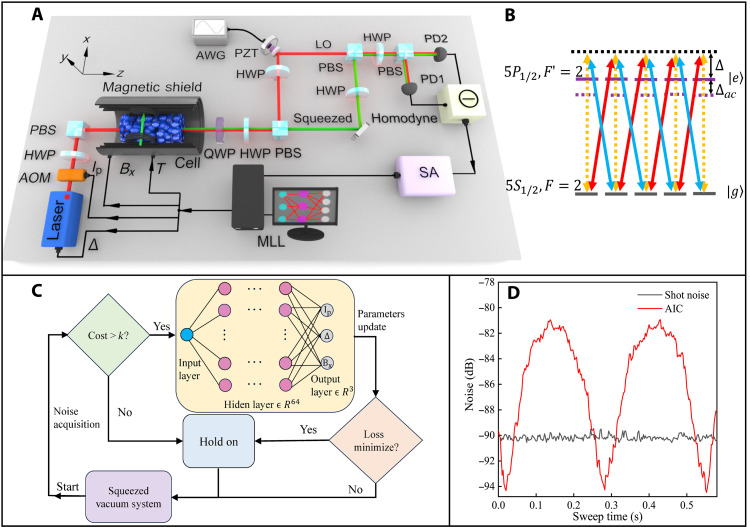
Experimental scheme and working principle. (**A**) Experimental setup. A pump laser enters into an ^87^Rb atomic vapor cell to generate squeezed vacuum light via polarization self-rotation (PSR) effect. AOM, acoustic-optic modulator; PBS, polarization beam splitter; HWP, half-wave plate; QWP, quarter-wave plate; AWG, arbitrary waveform generator; PZT, piezoelectric ceramics; LO, local oscillator; *I*_P_, Δ, *T*, and *B*_*x*_, the four optimized parameters; SA, spectrum analyzer; MLL, machine learning loop. (**B**) The atomic energy levels diagram of PSR squeezing. The pump and squeezing light both are resonant on the atomic transition from $|5^{2}S_{1/2},F=2\rangle$ to $|5^{2}P_{1/2},F\prime =2\rangle$. Δ and Δ_ac_ are the detuning frequency and ac-Stark shift of the pump field, respectively. The yellow dashed line shows linearly polarized pump light, a superposition of left (red solid line) and right (blue solid line) rotating circular polarizations. Atomic magnetic sublevels selectively absorb the light, causing PSR squeezing. (**C**) An MLL is used to artificially optimize the squeezing degree by dynamically controlling the frequency and intensity of the pump field, and magnetic field *B*_*x*_, which are parameters of atom-light coupling coefficient *g*. The parameters are updated by a NN containing an input layer, an output layer, and five hidden layers with 64 neurons in a single layer (see Methods in detail). (**D**) The 4.3-dB quadrature fluctuation of vacuum-squeezed light changes with the phase difference between the local oscillator (LO) and squeezed light. The intensity of LO is locked to 1 mW. The resolution bandwidth is 10 kHz, video bandwidth is 30 Hz, and the center frequency is 1 MHz.

The experimental setup is shown in Fig. 1A. A squeezed vacuum state is achieved only using one pump laser (*P*) in an ^87^Rb atomic vapor. The ^87^Rb atomic vapor cell (length, 7.5 cm; diameter, 2.0 cm) is placed inside magnetic shields to reduce the influence of environmental magnetic fields. A 15-mW *y*-polarized *P* field enters into the atomic cell along the *z*-axis direction. A signal (*S*) is generated via the PSR effect, whose polarization is perpendicular to the field *P*. After the cell, quadrature noise of the field *S* is detected using homodyne detection, where the intensity of the local oscillator is locked at 1.0 mW using a proportional-integral-derivative (PID) module. The noise is contingent upon four crucial parameters pertaining to the PSR effect, that is, the intensity and frequency of the pump field, atomic density, and magnetic field–induced splitting of the magnetic sublevel. In the experiment, iterative adjustments of *I*_P_, Δ, *T*, and *B*_*x*_ are necessary to achieve the maximum squeezing degree *Q*. The intensity *I*_P_ and detuning Δ of the pump field are controlled by an acoustic-optic modulator (AOM) and a frequency modulator of laser, respectively. Atomic density *n* is adjusted by the temperature *T* of the atomic cell via a temperature controller. The induced splitting of the magnetic sublevel is adjusted by an applied magnetic field *B*_*x*_, which is controlled via the electric current of the Helmholtz coil. The control mechanism is different for each parameter, but they can be adjusted synchronously by a programmable four-channel power supply.

### AIC operation

We use AIC for achieving automatic, optimal tuning of the four parameters, thereby implementing the generation of high-performance, stable squeezed light. The heart of AIC lies in the machine learning loop (MLL), whose operational principle is illustrated in Fig. 1C. This framework embodies a closed, condition-dependent feedback loop that interconnects a neural network (NN), a PSR-squeezed vacuum generation and measurement system, and a parameter modulation subsystem. MLL’s training and prediction are continuously updated, with each loop iteration lasting ~0.2 s. First, the MLL stochastically initializes parameters (*I*_P_, Δ, *T*, and *B*_*x*_) and transmits them to their respective controllers. The PSR system subsequently generates the field *S*, and its noise data are captured by a spectrum analyzer and fed back into the MLL module. Within the MLL, an NN performs sophisticated noise data analysis. The NN algorithm evaluates the current squeezing degree *Q* of the field *S* and instructs a new set of parameters to the controllers. Equipped with these updated parameters, the PSR system generates a new field *S* accompanied by fresh noise data, thereby completing a full iteration cycle of the MLL.

To achieve optimal squeezing, we designate *Q* as the cost function for the NN. The NN minimizes this cost value by using its built-in adaptive gradient descent algorithm. During each iteration, the *Q* value from the previous cycle is recorded and then compared against the predicted value generated by the NN. The mean square error between these two values serves as the loss function reflecting the maturity and progress of machine learning (see Methods). MLL iterates until the minimum loss is reached, indicating the achievement of the optimal squeezing degree and the algorithm training completion. Subsequently, the parameters are maintained until environmental disturbances cause a decline in *Q*, and then the MLL adjusts the parameters again within a small range to compensate for environmental interference and maintain the optimal squeezing state.

**Fig. 2.**
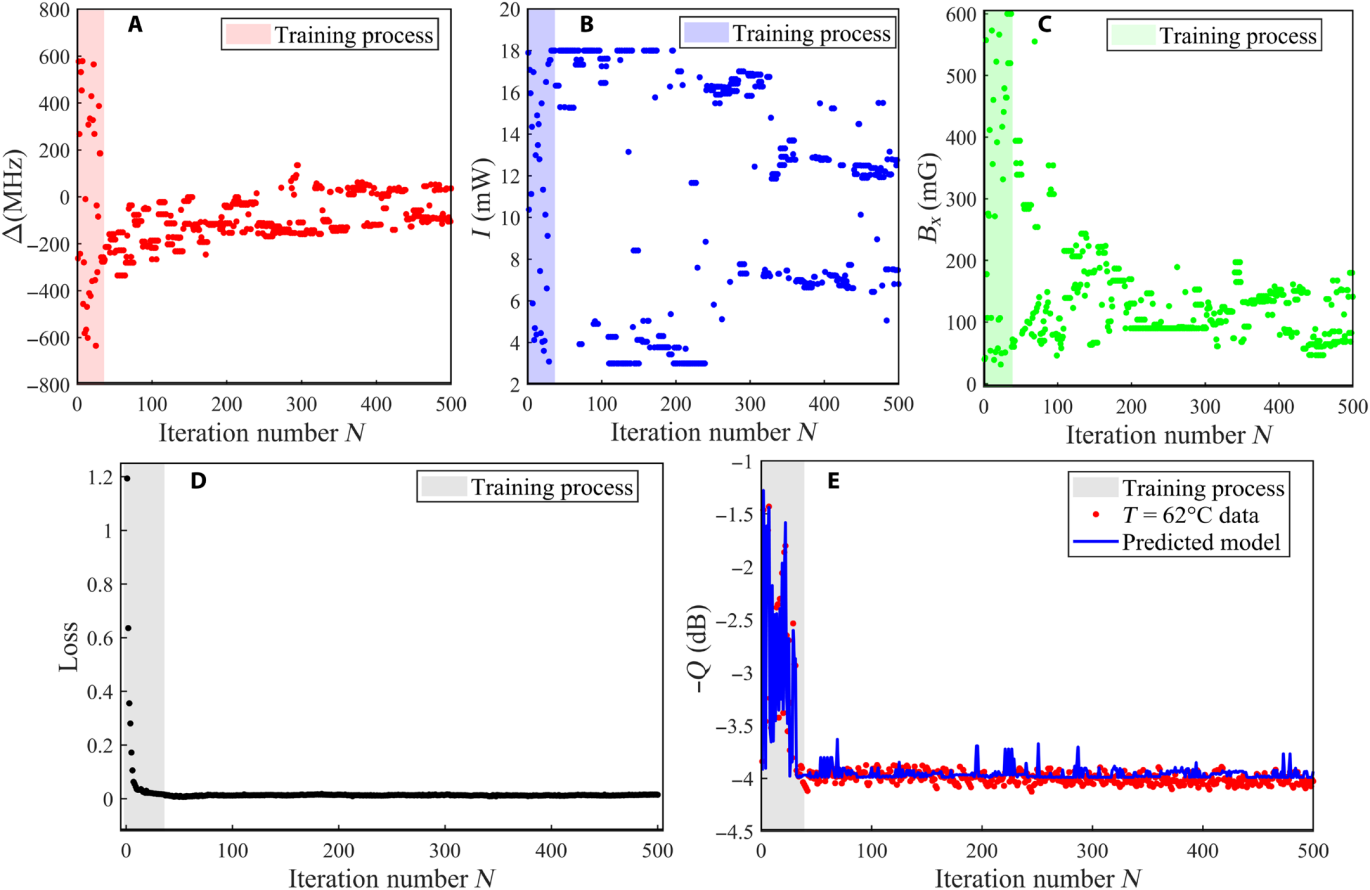
Experimental results of AIC real-time evolution with the iteration number *N*. (**A**) The detuning frequency Δ, (**B**) the intensity *I*_P_ of the field *P*, (**C**) the applied magnetic field *B*_*x*_, (**D**) the loss value, and (**E**) squeezing degree *Q*. All data are measured at atomic temperature *T* = 62.0°C.

To speed up machine learning, we predefined adjustment ranges for the four parameters from experience: *I*_P_ in [2 mW, 18 mW], Δ in [−700 MHz, 700 MHz], *T* in [38°C, 75°C], and *B*_*x*_ in [0 mG, 500 mG]. Given the slow pace of temperature adjustment in experiments, we initially identified the optimal temperature of 62.0°C using AIC and then fixed it to optimize the remaining three parameters dynamically. The parameters, squeezing degree, and loss value, plotted against the iterative number *N*, are illustrated in Fig. 2 (A to E). When *N* is below 35, notable fluctuations are observed in *I*_P_, Δ, and *B*_*x*_, while the NN’s loss function gradually decreases from 1.2 to near zero, signifying AIC’s learning processes and extensive exploration for optimal parameter configurations.

After 35 iterations, AIC begins to acquire the ability to adjust parameters intelligently. This enables the system to achieve and sustain an optimal state based on prior training. This is evident in Fig. 2E, where the optimal squeezing degree of 4.3 dB remains stable for a long time, despite random fluctuations in the three core parameters [as depicted in Fig. 2 (A to C) for *N* ≥ 35]. These fluctuations represent adaptive changes orchestrated by AIC to counteract environmental disturbances. Once environmental disturbances disrupt quantum squeezing, AIC intelligently adapts multiple PSR parameters in real time to keep the system operating at the optimal level.

### Multiparameter results and contributions

**Fig. 3.**
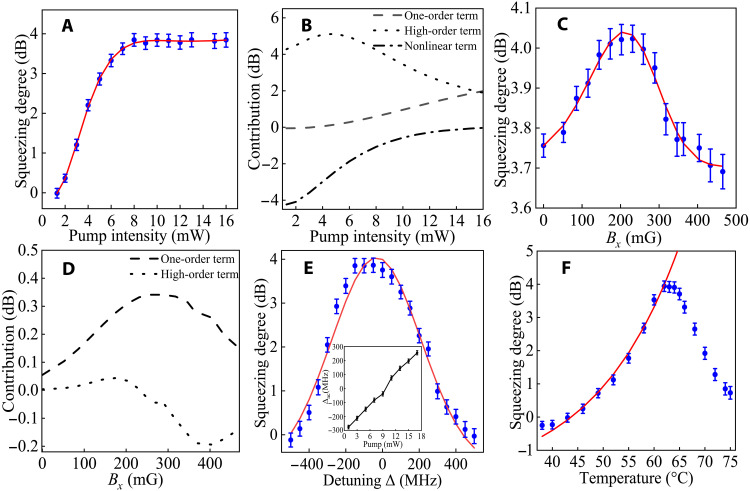
Multiparameter results and contributions. The squeezing degree as a function of the parameters, the pump intensity *I*_P_ (**A**), the applied magnetic field *B*_*x*_ (**C**), detuning frequency Δ of the pump field (**E**), and the temperature *T* of the atomic cell (**F**). The blue dots represent the experimental data, while the red solid line is the curve predicted by AIC. The experimental curves are obtained by changing one parameter and fixing the other three parameters. *I*_P_ = 15.0 mW, Δ = 100 MHz, *T* = 62.0°C, and *B*_*x*_ = 200 mG. Δ = 0 in (E) is the atomic transition from $|5^{2}S_{1/2},F=2\rangle$ to $|5^{2}P_{1/2},F\prime =2\rangle$. The dashed, dotted, and dash-dotted lines in (**B**) and (**D**) are the contributions of one-order, high-order, and nonlinear terms corresponding to the single-parameter predicted model in (A) and (C), respectively.

The experimental measurements have quantified the influence of *I*_P_, Δ, *T*, and *B*_*x*_ on the squeezing degree *Q*, with the results presented in Fig. 3. Subsequently, we conducted model prediction on the experimental data (see Methods). The fitting function for the squeezing degree *Q* (*I*_P_, Δ, *T*, and *B*_*x*_) incorporates one-order terms for *B*_*x*_ and Δ, second-order terms such as $B_{x}^{2}$ and $I_{P}^{2}$, and nonlinear terms including $\text{tanh}\left(\sqrt{B_{x}}\right)$ and $\text{tanh}\left(\sqrt{I_{P}}\right)$ among others. As a comparison, squeezing degree according to the conventional PSR theory (*40*–*42*) is given as

$$Q=20{\text{log}}_{10}\left(\mathrm{gl}/2\right)$$ (1)

where *g* is the atom-light coupling coefficient and *l* is the length of the atomic cell. *g* is the core parameter for *Q* with a fixed *l*. $g\propto \frac{\Delta }{\sqrt{I_{P}}}n\text{Tr}\left(\rho \hat{d}\right)$, where *n* is atomic density controlled by temperature *T*, ρ is the density matrix of the atomic magnetic energy level, and $\hat{d}$ is the atom dipole operator. The theoretical relationship between *Q* and these parameters exhibits a trend of monotonic increase or decrease. Below, we experimentally demonstrate the effect of these parameters on squeezing degree *Q* and compare the fitting function with the theoretical predictions in Eq. 1.

In Fig. 3A, *Q* increases with *I*_P_ until it reaches and remains the maximum value in the experiment. The fitting formula comprises a one-order *I*_P_ term, a second-order $I_{P}^{2}$ term, and a nonlinear $\text{tanh}\left(\sqrt{I_{P}}\right)$ term. The individual impacts of these terms on *Q* are depicted by dashed, dotted, and dash-dotted lines in Fig. 3B, respectively. The one-order term shows a monotonically increase aligning with the PSR theory trend, while the second-order and nonlinear terms bring decrease and saturation trends, and their contributions to *Q* are comparable to that of one-order terms. So in achieving high-performance squeezed states through strong atom-light coupling, the higher-order and nonlinear terms of *I*_P_ cannot be overlooked.

In Fig. 3C, it gives the relationship between *Q* and *B*_*x*_ $\left[\text{representing}\;\text{Tr}\left(\rho \hat{d}\right)\right]$. *Q* initially increases to a maximum value and then decreases with increasing *B*_*x*_, exhibiting a Gaussian-like shape. The fitting formula includes a one-order *B*_*x*_ term and a second-order term $B_{x}^{2}$. Two terms make considerable contributions to squeezing degree, and the nonlinear term has a certain negative effect, as shown in Fig. 3D. In this experiment, adjusting and measuring $\text{Tr}\left(\rho \hat{d}\right)$ pose challenges. A commonly used method to adjust it is by applying a magnetic field to the atoms. However, the influence of *B*_*x*_ on $\text{Tr}\left(\rho \hat{d}\right)$ is very complex, making it theoretically difficult to provide an analytical expression or even give numerical solutions to guide experiments. Despite we cannot assess consistency between theory and experimental results, the fitting function reveals that *B*_*x*_ has a non-monotonic and complex effect on the squeezing degree.

In Fig. 3E, with detuning frequency Δ, *Q* first increases to a maximum value and then monotonically decreases. The trend matches with the theory prediction, where the best squeezing degree appears at the atomic resonant frequency, but the frequency corresponding to the maximum squeezing degree differs by 100 MHz from the atomic resonance transition of $|5S_{1/2},F=2\rangle \to |5P_{1/2},F\prime =2\rangle$ in experiment. The frequency difference results from the ac-Stark effect of the pump field *P*, which can be confirmed in the inset of Fig. 3E. The frequency shift Δ_ac_ changes with the intensity of the field *P* linearly. Both of two parts, Δ_ac_ + Δ_P_, constitute the real detuning frequency. That is, adjusting *I*_P_ to optimize the squeezing degree simultaneously interferes total detuning of the PSR.

Figure 3F illustrates the variation of *Q* with respect to the atomic cell temperature *T*. Theoretically, based on the PSR model, temperature governs the atomic density *n* in a manner of continuous growth, and the squeezing degree should correspondingly increase with *T*. However, the experimental curve contradicts this prediction. Instead, the squeezing degree peaks at *T =* 62°C and subsequently decreases as *T* increases, primarily due to the absorption effect of atoms on the field *S*. Notably, this impact of atomic absorption on the squeezing degree is not accounted for in the conventional PSR theoretical model.

From the analysis of experimental data in Fig. 3, the inclusion of high-order and nonlinear dependencies of parameters substantially affects the squeezing degree peak. Conventional PSR theory overlooks interference between parameters and key experimental effects, leading to its inability to guide experiments effectively. AIC bridges the gap between concise theoretical models and intricate experimental observations by learning from squeezing data, particularly in the complex multiparameter regime crucial for generating extreme vacuum-squeezed light.

### Long-term stability

**Fig. 4.**
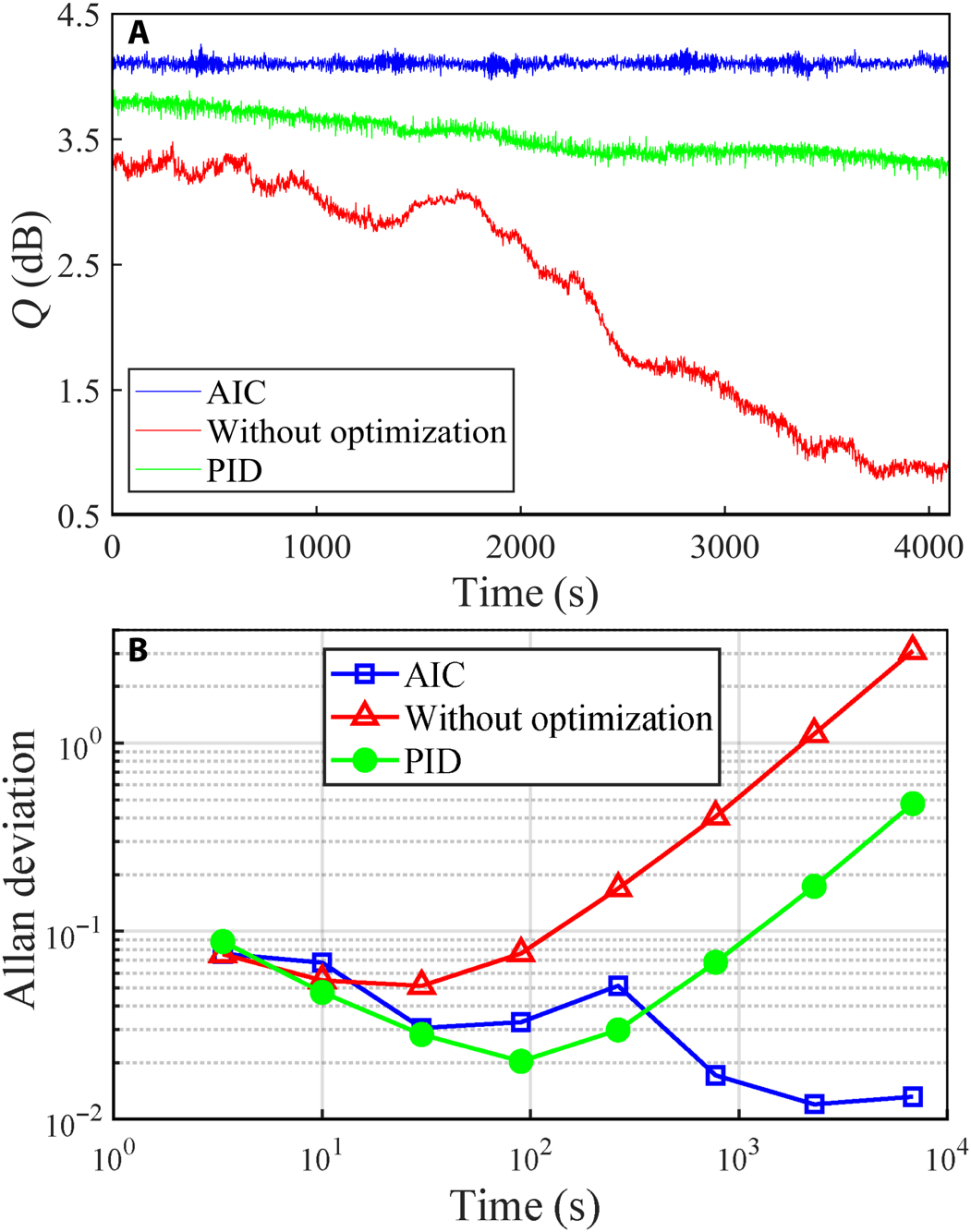
Long-term stability of AIC-assisted squeezed light. (**A**) Temporal evolution of the squeezed degree. (**B**) Allan deviation. The square, dot, and triangle lines represent squeezed light by AIC, PID control, and without any control, respectively. PID control locks the four parameters individually. Atomic temperature *T* is controlled at 62.0°C.

Figure 4 illustrates the environmental adaptability of the AIC-assisted squeezed light system. Figure 4A shows the squeezing degree within a 4000-s period after the system achieves optimal squeezed vacuum light. Using the AIC method, the squeezing degree remains consistently at 4.2 ± 0.1 dB, whereas, with conventional PID control, it gradually diminishes from 3.6 to 3.2 dB, and, without any feedback control, it drops substantially from 3.2 to 0.8 dB. This underscores the AIC system’s proficiency in adapting to real-time environmental and system fluctuations, intelligently tuning experimental parameters to sustain the squeezing state. The Allan variance is given in Fig. 4B to show the long-term stability of the squeezed state. The AIC method improves the stability time of the quantum squeezing system by more than one order of magnitude (from 100 to 4000 s) compared with PID control method because of its multiparameter optimization and self-monitoring (see Methods). The PID method only handles single-input, single-output relationships. For multiparameter problems, PID modules must lock parameters individually. In contrast, the AIC method adaptively optimizes multiparameters using its built-in NN. The adaptive correction of the optimal parameters predicted by the NN effectively improves the overall long-term stability of the squeezing system.

## DISCUSSION

We have developed an AIC method to surpass the constraints posed by the invalidation of conventional PSR theory in the strong coupling regime, enabling the preparation of high-quality squeezed light in atomic waveband. Using AIC, we achieve a maximum squeezing degree of 4.3 dB, which is equivalent to a lossless squeezing of 5.9 dB when accounting for 9.7% propagation loss and 6.5% detection loss. Notably, we sustain a stable 4.2 ± 0.1 dB squeezed light output for up to 2 hours, despite fluctuations in light intensity, frequency, and atomic temperature. The squeezing degree and stability achieved with our AIC method substantially outperform those attained using traditional PID control.

A further increase in the squeezing degree is possible by releasing additional mutually constrained system parameters, enhancing the complexity of the PSR mechanism while also providing more freedom for AIC-based optimization. For instance, our method uses a single laser for optical pumping and coupling light in atomic vapor, leading to a peak-then-dip squeezing trend with temperature changes, as depicted in Fig. 3F. By introducing a second laser to decouple these functions and optimizing the two additional parameters (frequency and intensity of the new laser) via AIC, we can expand more parameter dimensions, which will potentially improve the squeezing effect. This AIC method overcomes the bottleneck in achieving stable, high-performance squeezed light generation and provides a technique that does not rely on an exact theoretical model for manipulating complex atom-light systems. This approach is advantageous for PSR squeezing systems and conducive to developing other quantum systems, such as multimode entangled light, atomic clocks, tensor atomic magnetometers, and atomic spin squeezing systems. These systems generally face multiparameter complex bottlenecks and have high requirements for long-term stability.

## METHODS

### Artificial intelligence control

The AIC method is a self-adaptive loop in the presence of a time-varying environment. The whole AIC can be divided into three processes as follows in detail.

#### Initialization

The NN stochastically generates the beginning 30 group parameters within the parameter range set by us and synchronously records the corresponding quadrature-squeezing degree in the experiment, thus forming the initial training set. Here, we set the cost function of the NN

Cost=−Q (2)

where *Q* is the quadrature-squeezing degree of the squeezed vacuum light, and the NN will minimize the cost value according to the built-in adaptive gradient descent algorithm (*65*) during the training process.

#### NN search and prediction

We set *k* = −4.2 dB as a threshold for cost(*t*). When cost(*t*) is larger than the threshold *k*, the NN starts working. Based on the principle of minimization of the loss function, the NN updates the three parameters (*I*_P_, ∆, *B*_*x*_) for the next loop, according to the obtained cost value. To find the minimum cost value, the mean-square error loss function (*66*) is adapted in NN

$$\text{Loss}=\frac{1}{N}{\left[\text{cos}t_{\text{pre}}\left(t_{i}\right)-\text{cost}\left(t_{i}\right)\right]}^{2}$$ (3)

where *N* is the number of training samples, and cost_pre_(*t*_*i*_) and cost(*t*_*i*_) are the predicted value and the measured value of the *i* loop, respectively. When the loss function reaches the steady-state minimum, the NN will predict the intricate characteristic relationship between the squeezing degree and three parameters and obtain the optimized value.

#### Real-time monitoring

When the cost(*t*) < *k* condition is satisfied, the NN has found the optimal parameters. The system will dynamically stabilize at maximum squeezing. This process continuously monitors the squeezing degree of the PSR system. When the external environment’s factors such as temperature and magnetic field slow drift cause the steady-state conditions cost(*t*) < *k* to be broken, the NN will continue to update the parameters slightly in the previous optimal value nearby. This process effectively guarantees the robustness of interaction between the quantum system and NN, which leads to self-adapting to the external environment and keeps the long-term stability of the quantum system.

The training and prediction processes of MLL are dynamically updated, with each loop iteration lasting ~0.2 s due to a limitation of the AIC method: The system’s operational bandwidth must remain within the AIC’s bandwidth capacity. This bandwidth is constrained by the current electronic acquisition system, which includes the power supply, AOM modulator, and acquisition card. Future optimizations to the electronic acquisition system, aimed at achieving higher bandwidth and faster data rates, could substantially reduce iteration time without compromising optimization performance or multiparameter prediction accuracy. Such improvements would enhance the AIC’s operational bandwidth, thereby boosting its environmental adaptability and robustness in the generation of vacuum-squeezed light.

### Curve fitting and model prediction

The relationship between the squeezing degree *Q* and four parameters shows the saturation-type, simple Gaussian–type, Gaussian-like–type, and exponential-type model, respectively, as shown in Eq. 4 to 7. However, when dealing with multiparameter problems, things change, the NN prediction results show that there is an intrinsic correlation between the multiparameters, and they collectively exhibit a contribution to quantum squeezing

$$Q_{I_{P}}=-4.28e^{-\frac{{\left(-5.29+4.59\sqrt{I_{P}}+0.44I_{P}+0.02I_{P}^{2}\right)}^{2}}{2\times 4.73}}\;+4.26-0.058I_{P}+0.06\text{tanh}\left(\sqrt{I_{P}}\right)$$ (4)

QΔ=−0.74+4.78e−(Δ+38.2)22×243.52 (5)

$$Q_{T}=-2.0+1.915e^{0.06\left(T-43.1\right)}$$ (6)

$$Q_{B}=3.6+0.34e^{-\frac{{\left(B-262.8\right)}^{2}}{2\times 130.9^{2}}}$$ (7)

$$\begin{array}{cc} Q_{\text{multi}} & =10.9e^{-\frac{{\left(\Delta -0.38-0.09I_{P}^{1/2}+0.14I_{P}+0.04I_{P}^{2}-0.11B+0.2B^{2}\right)}^{2}}{2\times 0.63^{2}}}-6.89-0.30I_{P}\\ & +0.08I_{P}^{2}+0.29\text{tanh}\left(\sqrt{I_{P}}\right) \end{array}$$ (8)

Taking the case in Fig. 3 as an example, the multiparameter prediction result can be approximately written as the expression *Q*_multi_ (Eq. 8). To unify units, each parameter in Eq. 8 is normalized. It is clear that the experimental predicted formula deviates substantially from the theoretical formula (*40*–*43*) $Q\propto 20{\text{log}}_{10}\left[\frac{\Delta }{\sqrt{I_{P}}}n\text{Tr}\left(\rho \hat{d}\right)\right]$, where the atomic density $n\propto e^{T}$ approximately (*67*), and $\text{Tr}\left(\rho \hat{d}\right)$ is the average atom dipole. The theoretical curves of *Q* should have varied monotonically with ∆, *I*, and *T*, respectively, but experimental curve did not. The traditional PSR expression is accurate in weak coupling but disagrees with experiments in strong coupling due to higher-order effects and nonlinear correlations. Differently, the AIC method self-adaptively iterates multiparameters, which break through the scope of the weak interaction region of PSR and realize a higher squeezing. On the basis of AIC, we successfully predicted a more universal and accurate multiparameter model.
