# Supplementary material for: Ultra-stable and high-performance squeezed vacuum source enabled via artificial intelligence control
Source: Sci Adv. 2025 May 2;11(18):eadu4888. doi: 10.1126/sciadv.adu4888 (PMC12047443; doi:10.1126/sciadv.adu4888)
Supplement: Supplementary file 1 — Fig. S1 [file sciadv.adu4888_sm.pdf]

Science Advances
AAAS

Supplementary Materials for

Ultra-stable and high-performance squeezed vacuum source enabled via artificial intelligence control

Jie Zhao *et al.*

Corresponding author: Wenfeng Huang, wfhuang@phy.ecnu.edu.cn; Keye Zhang, kyzhang@phy.ecnu.edu.cn; L. Q. Chen, lqchen@phy.ecnu.edu.cn

Sci. Adv. **11**, eadu4888 (2025)
DOI: 10.1126/sciadv.adu4888

This PDF file includes:

Fig. S1

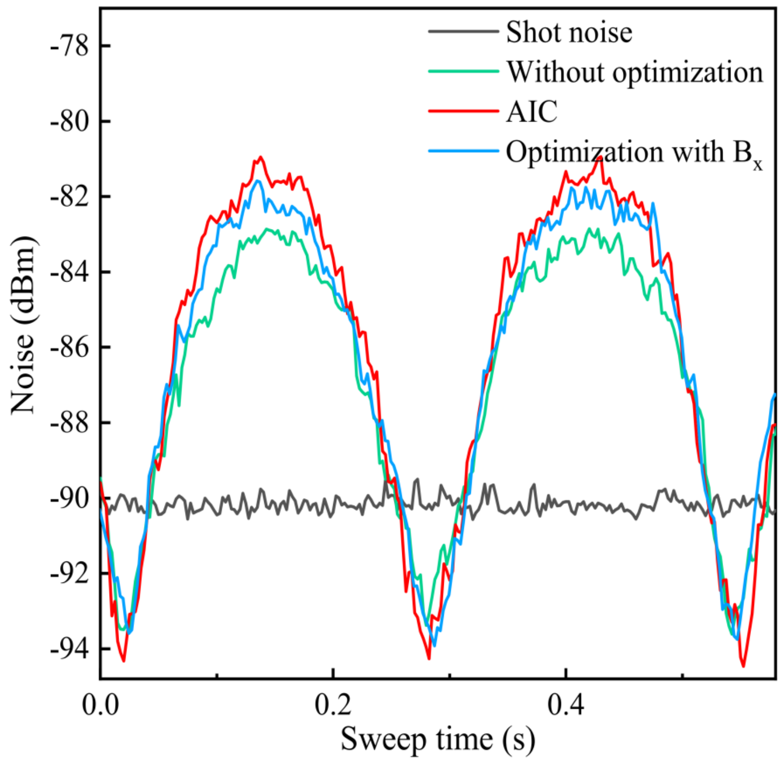

Fig. S1. Homodyne detection result. The figure compares PSR squeezing through the traditional method (without optimization), magnetic field optimization, and AIC dynamics method. The phase of the local light is scanned and the center frequency is 1 MHz.
